# Off-Label Use of Thrombopoietin Receptor Agonists: Case Series and Review of the Literature

**DOI:** 10.3389/fonc.2021.680411

**Published:** 2021-09-28

**Authors:** Marco Capecchi, Fabio Serpenti, Juri Giannotta, Loredana Pettine, Gianluigi Reda, Ida Martinelli, Andrea Artoni, Wilma Barcellini, Bruno Fattizzo

**Affiliations:** ^1^Department of Biomedical Sciences for Health, Università degli Studi di Milano, Milan, Italy; ^2^Fondazione IRCCS Ca’ Granda Ospedale Maggiore Policlinico, Angelo Bianchi Bonomi Hemophilia and Thrombosis Center, Milan, Italy; ^3^Department of Oncology and Onco-hematology, Università degli Studi di Milano, Milan, Italy; ^4^Fondazione IRCCS Ca’ Granda Ospedale Maggiore Policlinico, Hematology Unit, Milan, Italy

**Keywords:** thrombopoietin receptor agonist, eltrombopag, romiplostim, myelodysplastic syndromes, transplant, lymphoproliferative syndromes

## Abstract

Since their license in 2008, studies on thrombopoietin receptor agonists (TPO-RAs) are proceeding at a fast pace. Their favorable efficacy and safety profile makes them good candidates for the management of thrombocytopenia in different settings, even beyond their current indications. In the last 10 years, we faced patients with refractory thrombocytopenia that required treatment with off-label TPO-RA, despite the paucity of data in the literature and the possible risks, particularly that of thrombosis. We hereby report our 10-year real-life single-center experience of TPO-RA used off-label. Fourteen patients were divided into three groups according to the etiology of thrombocytopenia: myelodysplastic syndromes, post-transplantation, and lymphoproliferative diseases. Clinical features and results are reported within each group. Overall, TPO-RA proved effective in all these conditions achieving responses also in heavily pretreated patients. The overall response rate (ORR) was 100% in patients with thrombocytopenia after transplantation and in those with lymphoproliferative diseases and 75% in patients with myelodysplastic syndromes. The median duration of therapy was 285 days (range 93–1,513 days). Four patients (29%) discontinued treatment because of lack of response (n=2) or a sustained response (n=2). No grade 3–4 adverse events occurred, particularly no thrombosis. In our real-life experience, TPO-RAs were effective and safe and proved of value in the challenging management of patients with refractory thrombocytopenia associated with different conditions.

## Introduction

Since thrombopoietin receptor agonists (TPO-RAs) eltrombopag and romiplostim were licensed in the United States for treatment of immune thrombocytopenia (ITP) in 2008, their use has progressively increased, and they are currently available in more than 100 countries (1). Both TPO-RAs bind to the thrombopoietin (TPO) receptor, causing conformational change, activating the JAK2/STAT5 pathway, and resulting in higher megakaryocyte progenitor proliferation and increased platelet production. Indications for their use are similar in the United States and Europe. Eltrombopag (Revolade^®^, Promacta^®^) is approved for second-line therapy of ITP patients older than 1 year of age with a disease lasting at least 6 months (2), for second-line therapy of severe aplastic anemia after failure of immune suppressive therapy (3), and for the treatment of thrombocytopenia in adults with chronic hepatitis C treated with interferon-based regimens (4). Romiplostim (Nplate^®^) is only approved as second-line treatment for chronic ITP patients (i.e., after 12 months from diagnosis). Two other TPO-RAs have been recently approved, namely avatrombopag (Doptelet^®^) and lusutrombopag (Mulpleo^®^), specifically licensed for the treatment of patients with thrombocytopenia secondary to chronic liver disease undergoing invasive procedures (5, 6).

As for safety, the initial concerns on the risk of myelofibrosis induction have not been confirmed. Only a small number of patients develop moderate to severe reticulin and/or collagen fibrosis, which is usually reversible after drug discontinuation (7). A higher rate of venous thromboembolism has been reported, with a two- to threefold increased risk as compared with non-TPO-RA treated ITP patients (8, 9). Cataract and transiminitis are eltrombopag-associated adverse effects, whereas the development of neutralizing antibodies and pain after injection are associated to romiplostim (1).

Emerging evidence is being produced about TPO-RA efficacy and safety profile in different settings other than the registered ones. Overall, it appears relevant to understand which are the main clinical needs and the most promising fields in which to broaden TPO-RA use, given their convenient and safe profile. We hereby report our real-life single-center experience of TPO-RA use in unlabeled conditions and provide inherent literature review.

## Methods

We conducted an observational retrospective study to report the response rates and safety profile of TPO-RA used off-label. All consecutive patients aged >18 years and receiving out-of-label TPO-RA at our center between January 2010 and June 2020 were included. Patients were divided into three main groups according to the underlying cause of thrombocytopenia: post-transplant thrombocytopenia, myelodysplastic syndrome (MDS), and thrombocytopenia associated to lymphoproliferative diseases. All patients were assessed at baseline by whole blood counts with differential, peripheral blood smear, liver and kidney function tests, hemolytic markers (LDH, bilirubin, haptoglobin, reticulocytes), antiplatelet antibodies, and antiphospholipid antibodies and underwent a bone marrow evaluation with cytogenetic studies (karyotype for all patients and FISH for MDS patients), according to clinical practice. In MDS patients, a myeloid gene panel evaluating 69 genes implied in myeloid neoplasms had been performed at diagnosis in two patients; the test was unavailable for the others. Data on underlying disease, previous lines of therapy, drug dosing, discontinuation, bleeding/thrombotic events, and other adverse events were recorded. Clinical status and platelet response were recorded at 1, 3, 6, and 12 months after beginning of the drug. In patients with thrombocytopenia other than the ITP candidate to treatment with TPO-RA, a bone marrow evaluation at baseline is always performed. MDS patients receive further bone marrow assessments every 6 months and whenever clinically indicated. Patient monitoring and education for thrombotic complications and liver function tests evaluation were performed according to current clinical practice for ITP patients treated with TPO-RA. Response criteria were defined as partial response (PR) (platelet count >30,000) and complete response (CR) (platelet count >100,000). Adverse events were defined according to the CTCAE version 5.0.

## Results

**Figure 1:**
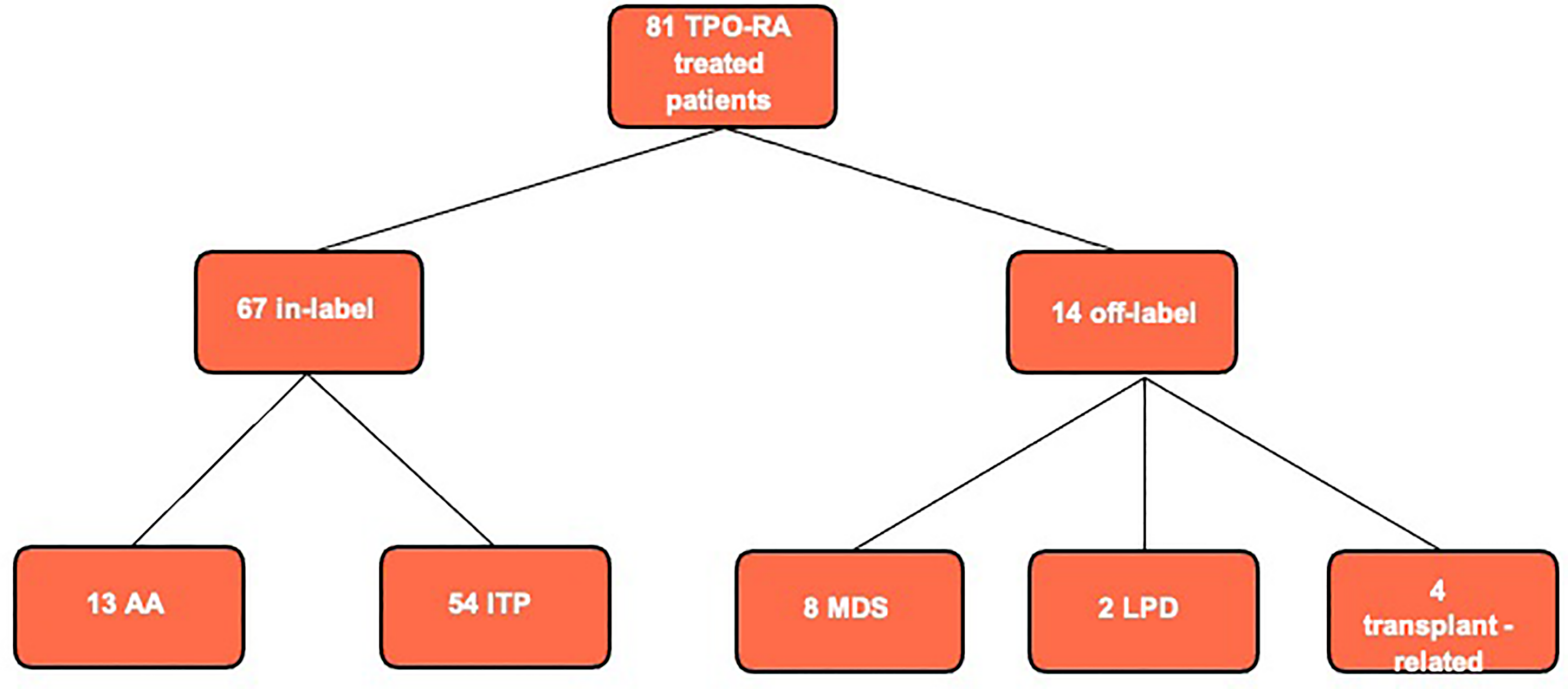
Flowchart of thrombopoietin receptor agonist (TPO-RA) treated patients at our center in the last 10 years. Aplastic anemia (AA) refers to aplastic anemia patients treated in second line after failure of immunosuppressive therapy as for current drug indications; we had no off-label AA patient treated in first line. MDS, myelodysplastic syndrome; LPD, lymphoproliferative disease; ITP, autoimmune thrombocytopenia.

**Figure 2:**
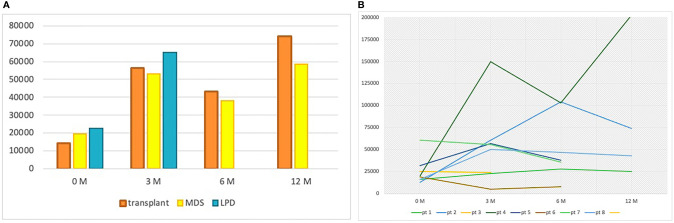
**(A)** Median platelet count at baseline, 3, 6, and 12 months per group of patients. At 6 and 12 months, no data are reported for the lymphoproliferative patients because the median follow-up was inferior to 6 months in this group. LPD, lymphoproliferative disease; MDS, myelodysplastic syndrome; M, months. **(B)** Platelet counts over time for each MDS patient.

From January 2010 to June 2020, a total of 81 patients have been treated with a TPO-RA (eltrombopag or romiplostim) at our referral center. A total of 67 patients received the TPO-RA for in-label conditions—ITP (54 patients) or aplastic anemia (13 patients)—while 14 patients received a TPO-RA for unlabeled conditions (**Figure 1** and **Table 1**). Among the latter group, four patients had a transplant-associated thrombocytopenia, eight suffered from MDS, and two had thrombocytopenia associated to a lymphoproliferative neoplasm. The median patient age was 62.5 years (range 36–86). All patients were treated with eltrombopag except for one who received romiplostim. The median duration of therapy was 285 days (range 93–1,513 days) with 4 patients (29%) discontinuing the drug. **Figure 2** shows median platelet trends over time among the different groups (**Figure 2A**) and platelet counts over time for each MDS patient (**Figure 2B**). Regarding responses to TPO-RA treatment, we only observed an increase in platelet counts, while no changes were recorded in erythrocytes and leukocytes in all the three clinical settings. No patient received blood transfusions or myeloid growth factors.

**Table 1:** Patient outcome stratified by group.

PATIENT ID	GROUP	NUMBER OF PREVIOUS THERAPY LINES	TPO-RA	MAXIMAL DOSE	DURATION OF THERAPY	RESPONSE	TIME TO RESPONSE	ADVERSE EVENT
T-01	TA-T	1	ELTROMBOPAG	150 mg die	181 d	PR	87	0
T-02	TA-T	1	ELTROMBOPAG	75 mg die	1513 d	PR	60	G1 liver
T-03	TA-T	2	ROMIPLOSTIM	6 mcg/kg/w	439 d	CR	119	0
T-04	TA-T	2	ELTROMBOPAG	50 mg die	93 d	CR	17	0
M-01	MDS	1	ELTROMBOPAG	75 mg die	702 d	PR	49	0
M-02	MDS	2	ELTROMBOPAG	75 mg die	1486 d	PR	57	0
M-03	MDS	3	ELTROMBOPAG	75 mg die	93 d	NR	-	PD to AML
M-04	MDS	3	ELTROMBOPAG	50 mg die	354 d	CR	14	0
M-05	MDS	1	ELTROMBOPAG	50 mg die	216 d	PR	40	0
M-06	MDS	2	ELTROMBOPAG	150 mg die	317 d	NR	-	0
M-07	MDS	4	ELTROMBOPAG	150 mg die	252 d	PR	25	0
M-08	MDS	1	ELTROMBOPAG	50 mg die	516 d	PR	44	0
L-01	LPD	4	ELTROMBOPAG	25 mg die	111 d	PR	20	0
L-02	LPD	2	ELTROMBOPAG	50 mg die	158 d	PR	139	0

Each row defines a patient. T-AT, transplant-associated thrombocytopenia; MDS, myelodysplastic syndrome; LPD, lymphoproliferative disease; TPO-RA, thrombopoietin receptor agonist; CR, complete response; PR, partial response; NR, non-response; G1, grade 1; PD to AML, progression to acute myeloid leukemia.

### Transplant Group

One patient received an autologous hematopoietic stem cell transplant (HSCT) due to an AL amyloidosis that was complicated by graft failure, leading to persistent thrombocytopenia in the post-transplant period. No more autologous stem cells were available to perform a second transplant procedure, and the patient was not suitable for an allogenic stem cell transplant given the comorbidities. After failure of danazol therapy, she was treated with eltrombopag at the same dose licensed for aplastic anemia (150 mg daily) reaching platelet transfusion independency at 3 months from TPO-RA start.

The other three patients of this group received a solid organ transplant (two liver and one kidney). The median time from transplantation to the onset of thrombocytopenia was 7 days for liver-transplanted patients. The kidney-transplanted patient had instead a late-onset thrombocytopenia (4 years and 2 months after transplantation) associated with graft rejection. Thrombocytopenia in these patients was interpreted as immune-mediated, but all failed steroid therapy. Two of them also received high-dose intravenous immunoglobulins, with only a transient PR in the kidney-transplanted patient. The median time from thrombocytopenia onset to TPO-RA therapy start was only 13 days (10–149 days), as the general clinical conditions of the patients required fast intervention to raise platelet counts. Two patients started eltrombopag 50 mg daily, and one of them had to increase the dose to 75 mg daily due to suboptimal response. The other patient was treated with romiplostim 1 mcg/kg per week, gradually increased to 6 mcg/kg per week to obtain a response. All patients responded, and median platelet counts increased from 8,000/mcl (range 2,000–30,000/mcl) pre-TPO therapy to 62,000/mcl (range 51,000–260,000/mcl), 91,000/mcl (range 43,000–139,000/mcl), and 74,000/mcl (range 64,000–84,000/mcl) at 3, 6, and 12 months, respectively (**Figure 2**).

The overall response rate (ORR) was 100% (1 CR and 3 PR). After 3 years of therapy, one patient who underwent liver transplantation stopped eltrombopag due to grade 1 hepatic toxicity together with a stable PR, which persisted even after TPO-RA discontinuation, until more than 6 months of follow-up (189 days). The median duration of therapy was 310 days (range 93–1,513), and overall survival (OS) was 100% with all patients being alive and in good general conditions at last follow-up visit.

### Myelodysplastic Syndrome Group

Eight patients received eltrombopag because of thrombocytopenic MDS. Six patients had low international prognostic scoring system (IPSS)/revised-IPSS, and the other two had intermediate-1 IPSS/revised-IPSS. Only one patient had abnormal cytogenetics with chromosome 8 trisomy. Mutational analysis with NGS was performed in two patients, one bearing a DNMT3A mutation with a variant allele frequency (VAF) of 3.6% and the other showing no detectable mutations. Five patients had a hypocellular bone marrow, three of them showing also reduced megakaryocytes. The other three patients showed an increased number of megakaryocytes, one of them with marked dysplastic features. Antiplatelet antibodies were found in five cases. The median number of therapy lines before TPO-RA was 2 (range 1–4). These included steroids (all patients, 5 PR), Cyclosporin A (3 patients, 2 PR), intravenous immunoglobulin (IVIG, 3 patients, 1 PR and 1 CR), danazol (3 patients, 2 PR), and rituximab (1 patient, transient PR). The median time elapsed from onset of thrombocytopenia to eltrombopag therapy was 1,582 days (range 30–5,878 days), and the median platelet count was 20,000/mcl (range 13,000–230,000/mcl) before therapy. Eltrombopag was started at 50 mg daily and raised according to clinical judgment, with a median steady dose of 75 mg (50–150 mg). The median platelet count was 53,000/mcl (range 5,000–150,000/mcl), 38,000/mcl (range 8,000–104,000/mcl), and 59,000/mcl (25,000–203,000/mcl) at 3, 6, and 12 months, respectively. ORR was 75% (5 PR and 1 CR). Three patients discontinued TPO-RA, two because of failure after 3 (93 days) and 10 months (317 days) of therapy, respectively. Interestingly, these patients were the only ones with intermediate-1 risk score according to IPSS/r-IPSS risk stratification, and both had an increased number of dysplastic megakaryocytes in their bone marrow biopsies. The other patient discontinued the drug due to persisting CR for more than 11 months (340 days). This patient experienced transient drops in platelet counts while tapering TPO-RA but was able to stop the drug keeping a sustained CR for 9 months (279 days). Thereafter, a relapse occurred again, successfully rescued with TPO-RA. No adverse events were observed in this group of patients. The median duration of therapy was 336 days (range 93–1,486 days) and the OS was 88%. The patient who died had a 15-year history of thrombocytopenic MDS heavily pretreated with steroids, IVIG, danazol, cyclosporine, and finally eltrombopag with no response. Three months after TPO-RA start, the patient evolved to AML, received induction chemotherapy and bone marrow transplantation, and thereafter died because of an infection complication. The long history of refractory disease and the short course of eltrombopag therapy before disease progression make it difficult to evaluate a possible cause–effect relationship between TPO-RA treatment and leukemic progression.

### Lymphoproliferative Group

Two patients had a lymphoproliferative disorder, one chronic lymphocytic leukemia (CLL) and one indolent non-Hodgkin lymphoma (not histologically classified due to the patient’s frailty and comorbidities that contraindicated biopsy).

The CLL patient had a long history (about 10 years) of indolent untreated disease before the development of thrombocytopenia. He received steroids, rituximab, rituximab-bendamustine, and rituximab-idelalisib all without efficacy. Thereafter, eltrombopag 25 mg daily was started, and platelets rose to 60,000/mcl after 3 months of therapy. At last follow-up, he was still on TPO-RA treatment, in good general conditions, and without need for further CLL therapy.

The indolent non-Hodgkin lymphoma patient presented to the emergency department with grade 4 thrombocytopenia (platelet count 1,000/ml) associated with bleeding (grade 3 lower gastrointestinal tract hemorrhage). Steroids and IVIG were started without any improvement in terms of transfusion needs. After 2 weeks, she was put on eltrombopag 50 mg daily with a PR allowing hospital discharge. At 3 months from TPO-RA start, the platelet count was 70,000/mcl without any bleeding symptom and no adverse event reported.

### TPO-RA Off Label: Available Literature

**Table 2** shows the most important trials and reports of TPO-RA use in a number of unlabeled conditions, including myeloid neoplasms (mainly MDS, but also acute myeloid leukemia and chronic myelomonocytic leukemia), postchemotherapy setting of solid tumors, secondary ITP (associated to both lymphoproliferative syndromes and systemic lupus erythematosus, SLE), graft failure after hematopoietic stem cell transplant, thrombocytopenia after heart and lung transplantation, non-HCV-related chronic liver diseases, and inherited thrombocytopenia (10–52). The majority of these studies are small trials, mostly phase I/II, with less than 50 patients enrolled. The heterogeneity of the study designs and endpoints of the trials makes it difficult to compare their results. Anyway, the overall response rates, reported accordingly to the study primary objective, were high and satisfactory (more than 50%) in most cases. Some discrepancies can be appreciated in the results for the myeloid malignancies group, characterized by high heterogeneity in disease type and severity, ranging from low- to high-risk MDS patients and to acute myeloid leukemia; furthermore, medications associated to TPO-RA may also confound the picture. Of note, none of the published or ongoing studies have assessed the use of TPO-RA in the liver or renal post-transplant setting, and none is a real-life study.

**Table 2:** Published and ongoing studies with TPO-RA used in unlabeled conditions.

**TRIAL**	**AUTHOR**	**STUDY DESIGN**	**DISEASE**	**N° OF PTS**	**DRUG**	**RESULTS^1^**
	Ramadan et al. (10)	phase I trial	CMML	7	E	ORR 42%
	Gudbrandsdottir et al. (11)	retrospective	miscellaneous	17	E/R	ORR 40%
NCT01481220	Svensson et al. (12)	phase I trial	hgh-risk MDS	12	E + A	ORR 75%
NCT01286038	Duong et al. (13)	phase I trial	high-risk MDS/AML	37	E	ORR 24%
NCT00903422	Platzbecker et al. (14)	phase I/II RCT	high-risk MDS/AML	98	E	ORR 28%*
NCT01440374	Mittelman et al. (15)	phase II RCT	high-risk MDS/AML	145	E	NA^
NCT01893372	Swaminathan et al. (16)	phase II RCT	hgh-risk MDS	28	E +/- A	ORR 11%
NCT02158936	Dickinson et al. (17)	phase III RCT	high risk MDS	356	A +/- E	ORR 16%*
NCT01890746	Frey et al. (18)	phase II RCT	AML	148	E	ORR 70%*
NCT00303472	Kantarjian HM et al. (19)	phase I/II trial	low-risk MDS	44	R	ORR 46%
NCT00303472	Sekeres et al. (20)	phase I/II trial	low-risk MDS	28	R	ORR 61%
2010-022890-33	Oliva et al. (21)	phase I/II RCT	low-risk MDS	90	E	ORR 47%
NCT00321711	Kantarjian et al. (22)	phase II RCT	low-risk MDS	40	R + A	NA^
NCT00418665	Wang et al. (23)	phase II RCT	low-risk MDS	39	R + L	NA^
NCT00321711	Greenberg PL et al. (24)	phase II RCT	hgh-risk MDS	29	R + D	NA^
NCT00614523	Kantarjian et al. (25)	phase II RCT	low-risk MDS	250	R	ORR 38%
NCT00472290	Fenaux et al. (26)	extension study	low-risk MDS	60	R	ORR 57%
NCT04324060	ongoing		low-risk MDS		E/R	
NCT01772420	ongoing		low-risk MDS		E + L	
NCT02912208	ongoing		low-risk MDS		E	
	Parameswaran R et al. (27)	retrospective	solid tumor chemotherapy	20	R	success rate 70%
	García Lagunar et al. (28)	retrospective	solid tumor chemotherapy	6	E	NA^
	Al-Samkari et al. (29)	retrospective	solid tumor chemotherapy	153	R	success rate 79%
NCT01147809	Winer et al. (30)	phase II RCT	solid tumor chemotherapy	26	E	success rate 86%
NCT01147809	Winer et al. (31)	phase II RCT	solid tumor chemotherapy	75	E	NA^
NCT02052882	Soff et al. (32)	phase II RCT	solid tumor chemotherapy	60	R	success rate 93%
NCT02227576	Le Rhun et al. (33)	phase II trial	solid tumor chemotherapy	20	R	success rate 60%
NCT04485416	ongoing	phase I trial	solid tumor chemotherapy		E	
NCT02093325	ongoing	phase III RCT	solid tumor chemotherapy		E	
	Shobha V et al. (34)	retrospective	TP secondary to SLE	12	E	ORR 100%
	Maroun MC et al. (35)	retrospective	TP secondary to SLE	3	E	ORR 100%
	González-López et al. (36)	retrospective	secondary ITP	87	E	ORR 38%
NCT01168921	Paul S et al. (37)	phase II trial	TP secondary to CLL	24	E	ORR 82%
NCT01610180	Visco et al. (38)	phase II trial	TP secondary to LPD	18	E	ORR 78%
NCT01610180	ongoing	phase II trial	TP secondary to CLL		E	
NCT01168921	ongoing	phase II trial	TP secondary to CLL		E	
NCT01610180	ongoing	phase II trial	TP secondary to LPD		E	
	Fu et al. (39)	retrospective	poor graft function after HSCT	38	E	ORR 63%
	Marotta et al. (40)	retrospective	poor graft function after HSCT	13	E	ORR 53%
	Tang et al. (41)	retrospective	poor graft function after HSCT	12	E	ORR 83%
	Tanaka et al. (42)	retrospective	poor graft function after HSCT	12	E	ORR 60%
	Samarkandi et al. (43)	retrospective	poor graft function after HSCT	21	E	ORR 75%
	Hartranft et al. (44)	retrospective	poor graft function after HSCT	13	R	ORR 53%
	Yuan et al. (45)	phase II trial	poor graft function after HSCT	13	E	ORR 62%
NCT01980030	Peffault de Latour et al. (46)	phase I/II trial	poor graft function after HSCT	24	R	ORR 66%
NCT03515447	Vourc’h et al. (47)	before - after study	TP in heart and lung transplant	20	R	NA^
NCT03437603	ongoing	phase II trial	poor graft function after HSCT		E	
NCT00861601	Kawaguchi et al. (48)	phase I/II trial	TP in iver disease - non HCV	8	E	ORR 48%
NCT01133860	Pecci et al. (49)	phase II trial	inherited (MYH9)	12	E	ORR 91%
NCT00909363	Gerrits et al. (50)	phase II trial	inherited (WAS)	8	E	ORR 62%
NCT02422394	Ongoing	phase II trial	inherited		E	
NCT04371939	ongoing	phase II trial	inherited (WAS)		E	
NCT03638817	ongoing	phase II trial	inherited		E	

Studies are grouped according to the indication for TPO-RA use. The number of trial as on ClinicalTrial.gov is reported when available. First author name is reported for published studies. RCT, randomized controlled trial; CMML chronic myelomonocytic leukemia; MDS, myelodysplastic syndrome; AML, acute myeloid leukemia; SLE, systemic lupus erythematosus; CLL, chronic lymphocytic leukemia; TP, thrombocytopenia; HSCT, hematopoietic stem cell transplantation; WAS, Wiskott–Aldrich syndrome; E, eltrombopag; R, romiplostim; A, azacytidine; L, lenalidomide; D, decitabine; ORR, overall response rate.

^1^Response rates are reported according to the primary endpoint of each study and therefore different rates are not comparable between studies. For trials on solid cancer chemotherapy, the term success rate more than ORR is applied, as main objectives were thrombocytopenia prevention and ability not to delay chemotherapy cycles.

^*^Experimental arm did not show better outcomes compared to placebo arm.

^^^Not applicable, due to different aims and outcome measures; among outcome measures: higher PLT counts/nadir, lower PLT transfusion rates, lower clinically relevant thrombocytopenic event (CRTE).

The most explored field of investigation for these drugs remains myelodysplastic syndromes, especially in the low-risk setting, where thrombocytopenia is common and can impact the quality of life of patients with a good prognosis. Eight trials have already been completed (19–26) and three are ongoing, including a phase II/III trial, with a cumulative ORR of 45%. Despite some older reports pointing out an increased leukemogenesis risk (53), a recent phase II trial (15) did not confirm these data and showed a reduction in the need of platelet transfusion support and bleeding episodes also in patients with high-risk MDS or AML treated with eltrombopag. Similarly, a phase I/II study (26) found romiplostim to be safe in low-risk MDS patients with a good response rate in terms of platelet count. The biggest study available in MDS is a phase III randomized controlled trial (RCT) (17) conducted in high-risk MDS patients with eltrombopag coupled to azacytidine. This trial failed to show any benefit of eltrombopag and was terminated prematurely.

Regarding chemotherapy-associated thrombocytopenia in solid tumors (27–33), TPO-RA reduced thrombocytopenic adverse events thus decreasing the delay between cycles and the need for chemotherapy dose reduction. A phase III RCT is currently ongoing on the use of eltrombopag for chemotherapy-induced thrombocytopenia in any solid tumor in Taiwan.

ITP secondary to SLE may not respond to disease-specific treatments (steroids, immunosuppressors, and biologics) and further impair patients’ quality of life. TPO-RA showed 100% response rates in a total of 15 SLE patients treated in two retrospective studies (34, 35).

ITP secondary to autoimmune and lymphoproliferative diseases (34–38) remains an understudied setting, with the majority of studies being retrospective and including a low number of patients, although with remarkable efficacy. Overall, 144 patients have been reported and the cumulative ORR was 57%. Indeed, thrombocytopenia refractory to first line steroids should be managed with lymphoma-specific treatment (e.g., chemo-immunotherapy in CLL) according to current guidelines.

Reports for the use of TPO-RA in inherited thrombocytopenia, such as MYH9-related disorders or Wiskott–Aldrich syndrome (49, 50), showed benefit on transfusion needs and hemostasis in a total of 80% of patients. There is also one report indicating benefit from TPO-RA in von Willebrand disease type 2B refractory to more conventional lines of therapy in emergency conditions (54).

Lastly, a promising field of interest, with an increasing number of reports in the last years, is the post- HSCT setting. TPO-RA could be of great benefit in prolonged cytopenia secondary to poor graft function that remains nowadays still difficult to manage and accounts for a significant proportion of nonrelapse mortality after transplant. Many recent retrospective series have reported encouraging results (39–46, 51, 52), with the biggest experience of the Spanish Group who reported an ORR of 72% in a cohort of 89 patients (51). Importantly, promising data exist in post-HSCT pediatric cohorts too (52).

## Discussion

In this study, we report the safety and efficacy of TPO-RA use outside approved indications in real-life cohort from a single referral center. These clinical settings represent the real life encountered by hematologists handling thrombocytopenia. We found that TPO-RAs are effective and manageable even in off-label settings, despite possible detrimental cofactors linked to underlying conditions such as cancer and transplant. Indeed, all the transplanted patients and those with lymphoproliferative syndromes responded after about 3 months of TPO-RA therapy, and among myelodysplastic patients, six out of eight achieved at least a PR.

Only 1 transplanted patient out of 14 treated with eltrombopag experienced a grade 1 hepatic toxicity, with complete recovery after drug discontinuation. No other TPO-RA-related adverse events were observed during the study.

Of note, one transplanted and one myelodysplastic patient maintained a stable response, partial and complete, respectively, after 189 and 279 days from TPO-RA discontinuation, showing that the achievement of a treatment-free remission is possible even in conditions other than ITP.

The favorable efficacy and safety profile emerging from our cohort is similar to that reported in the increasing number of studies addressing the use of TPO-RA in myeloid neoplasms (mainly MDS, but also acute myeloid leukemia and chronic myelomonocytic leukemia), in the postchemotherapy setting of solid tumors, in secondary ITP (associated to both lymphoproliferative syndromes or systemic lupus erythematosus), in graft failure after HSCT, in thrombocytopenia after heart and lung transplantation, in chronic liver diseases, and in inherited thrombocytopenia (**Table 2**). Thrombocytopenic low-risk MDS was the main clinical condition in which we used off-label eltrombopag. Compared to the biggest phase II trial of eltrombopag in low-risk MDS patients [EQol-MDS study (21)], we experienced a higher ORR (75% *vs* 47%) possibly due to the lower representation of intermediate-1 risk MDS in our cohort (25% *vs* 71%). Interestingly, the only two intermediate-1 risk patients were the ones who did not respond to eltrombopag.

An interesting point is that the employed doses of TPO-RA are extremely heterogeneous, reflecting the uncertainties in off-label conditions. Similarly, in this study, a wide range of doses of eltrombopag has been used, from 25 to 150 mg daily. However, both the lowest (25 mg) and the highest (150 mg) doses were associated with at least a PR, and the two CR occurred on eltrombopag at low dose (50 mg in both cases). This likely reflects the use of the off-label drug at the “minimal effective dose,” with the aim to limit toxicities, as currently suggested in ITP (55). On average, higher doses were requested in MDS-associated thrombocytopenia compared to transplant- and lymphoproliferative-associated thrombocytopenia, where the ITP dose (i.e., 50–75 mg daily) seems to be sufficient. This can be explained by the different pathogenesis of thrombocytopenia in these settings. In particular, post-transplant thrombocytopenia is the one that mostly resembles ITP, since the post-transplant period is characterized by a cytokine storm that may be responsible for the onset of cytopenia and may require several lines of immunosuppressive therapies other than steroids and IVIG (56). In contrast, thrombocytopenia associated with conditions that are characterized by a derangement of the myeloid stem cell, such as myelodysplasia, requires higher doses of TPO-RA to achieve a response. Indeed, TPO-RAs exert their stimulating/differentiating effect also on early hematopoietic stem cells (57); if the latter are dysplastic, the stimulus carried out by standard doses of TPO-RA may not be sufficient (58). As a matter of fact, doses as high as 300 mg daily have been used in RCT (15, 21). Clarifying the best TPO-RA schedule in MDS will be of great value, since 50% of these patients suffer from thrombocytopenia (59), which may be worsened by MDS therapy, and is mainly managed with transfusions and steroids.

The importance of bone marrow reserve is an issue also in lymphoproliferative diseases, where the use of TPO-RA induces an overall response of about 40–50% (58), which increases to 70–80% (the same of primary ITP), if considering only true immune-mediated thrombocytopenia.

Thrombotic events, although infrequent, have been reported in ITP patients treated with TPO-RA with an incidence rate ranging from 1.4 to 4.3 per 100 patient-years (1); if we consider the conditions other than ITP, the majority of studies reported an incidence of venous thromboembolism lower than 2% (range 0–1.6%), while higher rates (≥2%) were reported in MDS/AML and postchemotherapy ITP (conditions *per se* associated with increased thrombotic risk), possibly related to the higher doses of TPO-RA used. Consistently, despite the heterogeneity of available trials, a trend toward increased thrombosis frequency is noted with higher TPO-RA doses; and almost all studies using eltrombopag > 200 mg reported at least one thrombotic event. In our study, no patients developed a thrombotic event, neither venous nor arteriosus. Even if the small number of patients does not allow taking definitive conclusions, the long follow-up and the supramaximal doses of TPO-RA employed in some patients suggest that the risk of thrombosis is not significantly increased.

Finally, some concerns about the potential clonal evolution induced by a sustained stem-cell stimulation under TPO-RA have been raised, particularly in aplastic anemia and MDS. However, in the prospective randomized EQoL-MDS study, leukemic evolution was comparable among the two arms (12% in eltrombopag *versus* 16% in the placebo arm), even if the long-term assessment is still ongoing (21). In our study, only one patient with a long history of heavily pretreated MDS presented a rapid evolution to acute leukemia (3 months after start of eltrombopag), making it difficult to evaluate a possible cause–effect relationship between TPO-RA treatment and leukemic progression. All the other patients were on long-term TPO-RA (range 216–1,486 days) without signs of evolution.

## Conclusions

Our real-life report of TPO-RA off-label use, even though on a small number of patients, highlights their efficacy and safety in difficult-to-manage thrombocytopenia forms, including post-transplant ITP and cases secondary to MDS and lymphoproliferative syndromes. Despite the awareness on thrombotic risk and on the possible clonal evolution should remain high, TPO-RA use in these conditions may have a significant impact on clinical management.

## Data Availability Statement

The raw data supporting the conclusions of this article will be made available by the authors, without undue reservation.

## Ethics Statement

The studies involving human participants were reviewed and approved by Comitato Etico Milano Area 2. The patients/participants provided their written informed consent to participate in this study.

## Author Contributions

MC and FS equally contributed to the manuscript. MC and BF designed the study. FS, JG, LP, and GR assessed the patients for eligibility, collected data, and critically revised the manuscript. MC, FS, BF, IM, AA, and WB contributed to developing the manuscript, and drafting and revising the text, tables, and figure. All authors contributed to the article and approved the submitted version.

## Funding

This work was supported by the Italian Ministry of Health—Bando Ricerca Corrente.

## Conflict of Interest

MC reports nonfinancial support from Roche, Novonordisk, and Sobi and honoraria from Daiichi Sankyo. AA reports nonfinancial support from Bayer and Roche and honoraria from Janssen outside of the submitted work. IM reports personal and nonfinancial support from Bayer and Roche outside of the submitted work. WB received consultation honoraria from Novartis, Apellis, Alexion, Agios, and Sanofi. BF received consultation honoraria from Amgen, Novartis, and Momenta.

The remaining authors declare that the research was conducted in the absence of any commercial or financial relationships that could be construed as a potential conflict of interest.

## Publisher’s Note

All claims expressed in this article are solely those of the authors and do not necessarily represent those of their affiliated organizations, or those of the publisher, the editors and the reviewers. Any product that may be evaluated in this article, or claim that may be made by its manufacturer, is not guaranteed or endorsed by the publisher.
